# Complement Factor H-Related Protein 4A Is the Dominant Circulating Splice Variant of *CFHR4*

**DOI:** 10.3389/fimmu.2018.00729

**Published:** 2018-04-17

**Authors:** Richard B. Pouw, Mieke C. Brouwer, Anna E. van Beek, Mihály Józsi, Diana Wouters, Taco W. Kuijpers

**Affiliations:** ^1^Department of Immunopathology, Sanquin Research and Landsteiner Laboratory of the Academic Medical Center, University of Amsterdam, Amsterdam, Netherlands; ^2^Department of Pediatric Hematology, Immunology and Infectious Diseases, Emma Children’s hospital, Academic Medical Center, Amsterdam, Netherlands; ^3^MTA-ELTE “Lendület” Complement Research Group, Department of Immunology, ELTE Eötvös Loránd University, Budapest, Hungary; ^4^Department of Blood Cell Research, Sanquin Research and Landsteiner Laboratory of the Academic Medical Center, University of Amsterdam, Amsterdam, Netherlands

**Keywords:** factor H-related-4A, factor H-related-4B, factor H, factor H-related proteins, CFHR4 gene, the complement system

## Abstract

Recent research has elucidated circulating levels of almost all factor H-related (FHR) proteins. Some of these proteins are hypothesized to act as antagonists of the important complement regulator factor H (FH), fine-tuning complement regulation on human surfaces. For the *CFHR4* splice variants FHR-4A and FHR-4B, the individual circulating levels are unknown, with only total levels being described. Specific reagents for FHR-4A or FHR-4B are lacking due to the fact that the unique domains in FHR-4A show high sequence similarity with FHR-4B, making it challenging to distinguish them. We developed an assay that specifically measures FHR-4A using novel, well-characterized monoclonal antibodies (mAbs) that target unique domains in FHR-4A only. Using various FHR-4A/FHR-4B-specific mAbs, no FHR-4B was identified in any of the serum samples tested. The results demonstrate that FHR-4A is the dominant splice variant of *CFHR4* in the circulation, while casting doubt on the presence of FHR-4B. FHR-4A levels (avg. 2.55 ± 1.46 µg/mL) were within the range of most of the previously reported levels for all other FHRs. FHR-4A was found to be highly variable among the population, suggesting a strong genetic regulation. These results shed light on the physiological relevance of the previously proposed role of FHR-4A and FHR-4B as antagonists of FH in the circulation.

## Introduction

**Figure 1:**
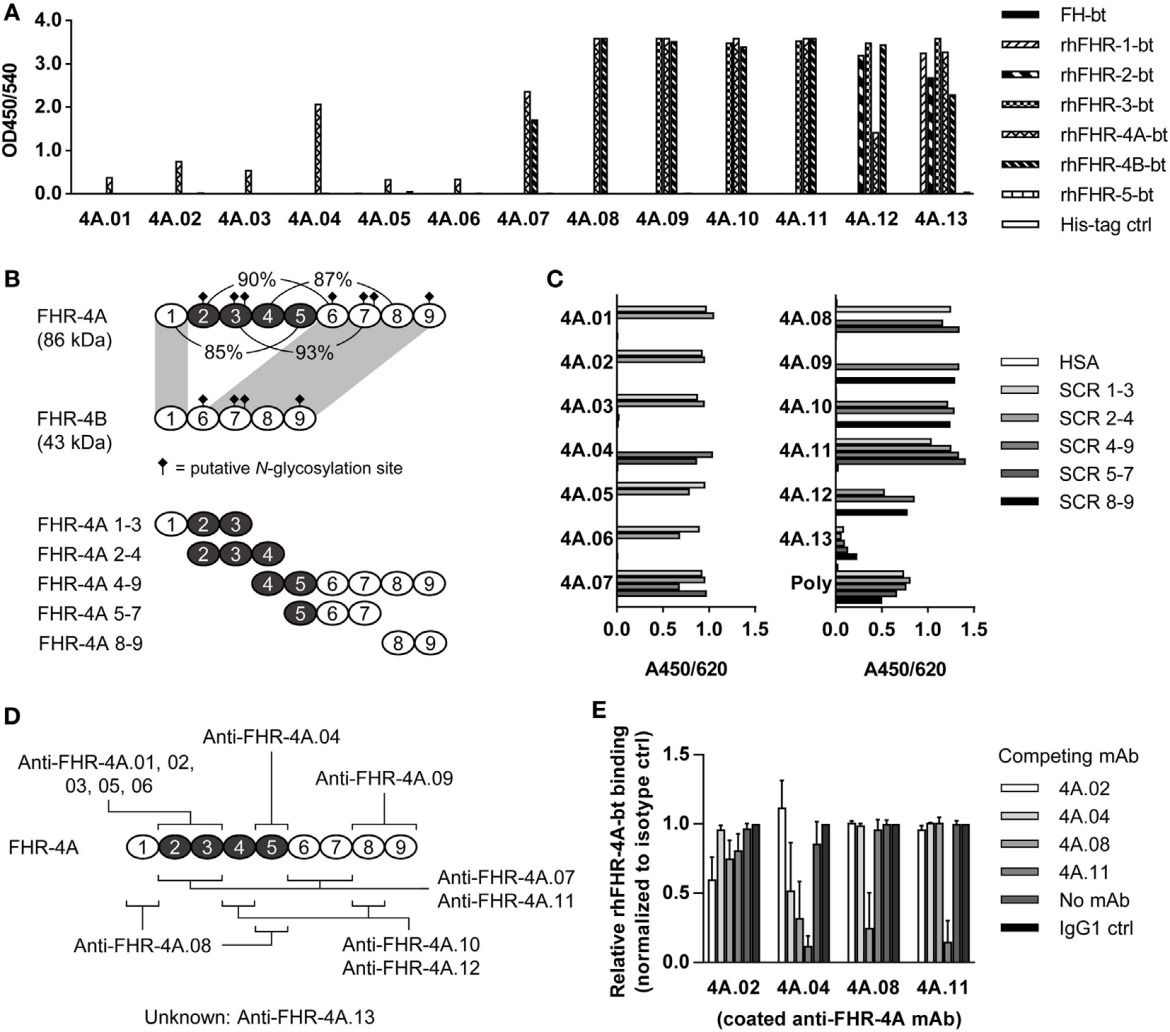
Characterization of anti-FHR-4A monoclonal antibodies (mAbs). **(A)** Cross-reactivity of the anti-FHR-4A mAbs to biotinylated rhFHR proteins and biotinylated plasma-derived FH was determined by ELISA. **(B)** Schematic representation of FHR-4A, FHR-4B, and the recombinant fragments of FHR-4A used for epitope mapping, with the duplicated SCR domains in FHR-4A depicted in black. Corresponding percentage of sequence identity between the domains in FHR-4A is indicated. Domains 1 and 6–9 of FHR-4A are completely identical to the domains of FHR-4B (indicated by gray shading). **(C)** Epitope mapping of anti-FHR-4A antibodies using the fragments of rhFHR-4A as depicted in **(B)**, determined by ELISA. As a control, polyclonal anti-FHR-3 (poly), which cross-reacts with all FHR proteins was used. **(D)** Schematic representation of the epitope location of each of the anti-FHR-4A mAbs. Single epitope mAbs are listed above FHR-4A, mAbs with multiple epitopes below. Note that most cross-reactive mAbs have two epitopes in FHR-4A, due to the high sequence similarity of the SCR domains. **(E)** Competition ELISA with monospecific mAbs (anti-FHR-4A.02 and 4A.04) and cross-reactive mAbs (anti-FHR-4A.08 and 4A.11). Binding of biotinylated rhFHR-4A is expressed as relative to the binding of biotinylated rhFHR-4A without any competing mAb. Bars represent mean of independent replicates with error bars indicating SD. All graphs are representative of multiple independent experiments.

The complement system is an evolutionarily ancient protein cascade which, through a series of events, recognizes, attacks, and kills foreign cells like bacteria, but can also target host cells [reviewed by Ricklin et al. (1)]. In order to prevent damage of healthy host cells, humans possess several complement regulators. Some are membrane bound and expressed on the cell surface; however, one of the most important regulators, complement factor H (FH) circulates freely in plasma. FH is a 155 kDa glycoprotein that circulates in blood with a reported average concentration ranging from 233 up to 400 µg/mL or 1.5–2.6 µM (2–6). FH is a crucial regulator of the alternative activation pathway of the complement system, orchestrating complement activation toward foreign cells by specifically binding to and inhibiting complement on human cells. FH belongs to the FH protein family, which consists of eight proteins derived from six genes, encoded in tandem in the *CFH* locus. The *CFH* gene itself encodes two proteins, FH and its splice-variant FH-like 1 (FHL-1). Next to FH and FHL-1, six factor H-related (FHR) proteins are found in plasma. Like FH and FHL-1, the FHRs are completely comprised of domains called short consensus repeat (SCR) domains. FHR-1, FHR-2, FHR-3, and FHR-5 are encoded separately by corresponding genes (*CFHR1, CFHR2, CFHR3*, and *CFHR5*), while FHR-4A and FHR-4B are splice variants encoded by *CFHR4* (7, 8). FHR-4A is the largest FHR (86 kDa) and comprises nine SCR domains, while FHR-4B has been predicted to consist of five SCR domains (43 kDa) (7, 8). The five SCR domains of FHR-4B are completely identical to SCR 1 and SCRs 6–9 of FHR-4A (Figure 1B). The unique four SCR domains between SCRs 1 and 6 of FHR-4A appear to have arisen from an internal duplication in *CFHR4* and are highly similar to the other SCR domains in FHR-4A and, therefore, also to FHR-4B (8). SCR 1 is highly similar to SCR 5 (85%), 2–6 (90%), 3–7 (93%), and SCR 4 to SCR 8 (87%) in FHR-4A. This makes it challenging to specifically distinguish FHR-4A from FHR-4B in immunoassays.

It is still unclear what the role of FHR-4A and FHR-4B is within the complement system. Increasing evidence indicates that FHRs act as antagonists of FH, competing with FH for the binding to complement C3b and human cell surfaces [reviewed by Józsi et al. (9)]. FHR-4A and -4B seem to lack physiologically relevant complement inhibitory activities on their own. FHR-4A has been reported to enhance the co-factor activity of FH at supraphysiological concentrations (10, 11). Furthermore, binding of FHR-4A and FHR-4B instead of FH allows complement activation to occur on the surface (11) and FHR-4A and FHR-4B might act as a competitor of FH. This form of regulation might serve to fine-tune complement inhibition by FH on surfaces where balanced complement activation is required for clearance, such as on necrotic and apoptotic cells. This hypothesis would also explain associations between genetic variation in *CFHR* genes and various diseases. For instance, the lack of *CFHR3* and *CFHR1* due to copy number variation (CNV) has been reported to be protective against age-related macular degeneration (12, 13). However, no such link with disease has yet been found for *CFHR4*. Like other FHRs, FHR-4A and FHR-4B bind to C3b and C-reactive protein, allowing complement activation to occur (11, 14, 15). Furthermore, FHR-4A has been reported to recruit CRP to necrotic cells *in vitro*, allowing more complement activation to occur, and is found to be accumulated in necrotic tissue (14). However, in contrast to all other members of the FH protein family, FHR-4A and FHR-4B seem to lack any affinity toward heparin (16, 17).

Highly relevant for the proposed antagonistic properties of FHR proteins is their physiological concentration, especially in relation to FH. Recently, we have reported normal protein concentrations for all FHRs in >100 healthy individuals, except for FHR-4A and FHR-4B. FHR-1/1 homodimers, FHR-2/2 homodimers, and FHR-1/2 heterodimers circulate at average concentrations of 14.6 (±3.0), 0.7 (±0.4), and 5.8 (±2.4) μg/mL, respectively, in healthy donors with two *CFHR1* copies, whereas FHR-5/5 homodimers circulate at 1.7 (±0.4) μg/mL in healthy donors (18). Of note, the FHR-1 total levels have recently also been reported to be 122 (±26) μg/mL, without distinguishing between homo- or heterodimers, nor fully describing the calibration used (19). However, a third, independent, group recently reported combined, total FHR-1, FHR-2, and FHR-5 levels, measured in an immunoassay, to be 10.7 (±5.4) μg/mL, thus supporting the lower levels for FHR-1 and FHR-2 (20). FHR-3 circulates at average concentrations of 0.7–1.1 µg/mL (6, 21). For FHR-4A and FHR-4B, only total FHR-4 levels ranging from 6.5 to 53.9 µg/mL with an average concentration of 25.4 µg/mL have been described, measured in only 11 healthy individuals (11). These levels were determined without distinguishing between FHR-4A and FHR-4B. Therefore, in order to specifically measure FHR-4A and FHR-4B, as well as establishing normal levels in a larger cohort of healthy individuals, we have developed specific reagents for FHR-4A and FHR-4B. Monoclonal antibodies (mAbs) were used to specifically detect FHR-4A, but we were unable to detect any freely circulating FHR-4B in human serum, despite the use of various cross-reactive mAbs. Finally, we demonstrate that FHR-4A levels vary greatly among healthy individuals.

## Materials and Methods

### Samples

Healthy donor serum samples were collected as part of a previous study from anonymous, healthy volunteers with informed, written consent in accordance with Dutch regulations and this study was approved by the Sanquin Ethical Advisory Board in accordance with the Declaration of Helsinki (6). Normal human serum (NHS) pool comprises serum from 400 healthy donors. FHR-3-deficient serum pool comprises serum of four healthy donors previously genotyped by multiplex ligation-dependent probe amplification to carry no *CFHR3* gene copies (6). Samples of patients with confirmed bacterial infections were collected as part of the EUCLIDS project (van Beek et al., manuscript in preparation). CRP levels were determined in these samples as part of routine testing.

### Proteins and Reagents

Rat anti-mouse kappa (RM-19) mAb was from Sanquin Reagents (Sanquin, Amsterdam, the Netherlands). High-performance ELISA buffer (HPE) was provided by Sanquin. Proteins were biotinylated according to the manufacturer’s instructions using EZ-Link Sulfo-NHS-LC-Biotin, No-Weigh Format (Thermo Scientific, Waltham, MA, USA), when indicated. Polyclonal anti-FHR-3 antibodies and mAb clone anti-FHR-3.3 were obtained and characterized as part of a previous study (6). Recombinant human (rh) FHR proteins, containing a C-terminal 6×-histidine tag, were produced and purified as previously described (6). In short, proteins were expressed by transient transfection of pcDNA3.1 expression vectors in HEK293F cells, after which proteins were purified from the supernatant by Ni^2+^ affinity chromatography using HisTrap™ High Performance 1 mL columns (GE Healthcare Life Sciences, Freiburg, Germany). rhFHRs were filtered and concentrated using Amicon^®^ Ultra Centrifugal Filter Devices (Merck Millipore, Darmstadt, Germany) according to the manufacturer’s instructions using appropriate molecular weight cut-offs. For rhFHR-4A, a 100 kDa cut-off Amicon^®^ Filter was used to further purify rhFHR-4A from any high molecular weight aggregates observed after Ni^2+^ affinity chromatography purification (Figures S1A,B in Supplemental Material).

### Immunization, mAb Generation, and Characterization

Anti-FHR-4A mAbs were generated, screened, and purified as previously described, using rhFHR-4A as immunogen (6). Isotypes of mAbs were determined by ELISA or with the use of the Mouse mAb Isotyping Kit (Hycult Biotech, Uden, the Netherlands) according to the manufacturer’s instructions. Cross-reactivity against other FH protein family members was determined as previously described (6).

### Epitope Mapping

The epitope location of each of the anti-FHR-4A mAbs was determined using rhFHR-4A, rhFHR-4B, and rhFHR-4A fragments consisting of SCR domains 1–3, 2–4, 4–9, 5–7, or 8–9 as previously described (15). Proteins and fragments were coated (4 µg/mL in PBS) onto Nunc Maxisorp 96-well microtiter plates (Invitrogen, Life Technologies, Carlsbad, CA, USA) by overnight (O/N) incubation at 4°C. Plates were washed three times with PBS + 0.02% (w/v) Tween-20 (PT) and blocked with 4% (w/v) BSA in PBS by incubation for 1 h. After another wash with PT, wells were incubated with the anti-FHR-4A mAbs diluted in PT for 1 h. Unbound anti-FHR-4A mAbs were washed away by washing the wells five times with PT, followed by incubating with 0.05% (v/v) HRP-conjugated rabbit anti-mouse IgG (Dako Agilent, Santa Clara, CA, USA) for 1 h and subsequently washing the plates five times. The ELISA was developed using 3,5,3′,5′-tetramethylbenzidine (TMB) solution and the reaction was stopped using 50 µL 2 M H_2_SO_4_. As a positive control, FHR-4A fragments were detected using polyclonal rabbit anti-FHR-4A, followed by HRP-conjugated goat anti-rabbit IgG (Dako Agilent). All ELISA steps were performed with a final volume of 50 µL per well and incubated at room temperature while shaking unless stated otherwise. Absorption was measured at 450 nm and corrected for background absorbance at 620 nm.

### Competition ELISA

To determine whether the mAbs competed for the binding of rhFHR-4A, Nunc Maxisorp 96-well microtiter plates (Invitrogen) were coated with 100 µL of 2 µg/mL anti-FHR-4A mAbs, in PBS, by incubating O/N at room temperature. Next, the plates were washed with PT. Biotinylated rhFHR-4A (0.1 µg/mL, in HPE) was incubated with 10 µg/mL of each mAb for 20 min, followed by incubation on the washed plate for 1 h. Next, unbound biotinylated rhFHR-4A was washed away and the wells were incubated with 0.01% (v/v) strep-poly-HRP (Sanquin), diluted in HPE, for 20 min. After washing, the assay was developed by addition of 100 µL of 100 µg/mL TMB in 0.11 M sodium acetate containing 0.003% (v/v) H_2_O_2_, pH 5.5. Substrate conversion was stopped after approximately 10 min by addition of 100 µL 2 M H_2_SO_4_. Absorbance was measured at 450 nm and corrected for the absorbance at 540 nm with a Synergy 2 Multi-Mode plate reader (BioTek Instruments, Winooski, VT, USA). All ELISA steps were performed with a volume of 100 µL per well and incubated at room temperature while shaking unless stated otherwise.

### Sucrose Gradient

Sucrose gradients were described previously (18). In short, NHS or FHR-3-deficient serum (150 µL of 50%, v/v, pooled serum, diluted in PBS) were loaded on 5–32.9% (w/v) sucrose (Merck 1.07654) gradients. Gradients were centrifuged for 20 h at 160,000 × *g* after which they were fractionated in 24 fractions of 500 µL. Proteins were immunoprecipitated and visualized on Western Blots as described below.

### Immunoprecipitation (IP)

Factor H-related-4A and FHR-4B were immunoprecipitated from human healthy donor serum or sucrose gradient fractions using indicated mAbs. IP was performed by incubating 200 µL serum or 250 µL sucrose gradient fraction with 500 µL of 5 mg/mL CNBr-activated sepharose (GE Healthcare, Little Chalfont, UK) to which RM-19 was coupled (25 mg mAb per 1 g sepharose), and 50 µL of 100 µg/mL mAb, diluted in PBS supplemented with 0.1% Tween-20, 0.1% BSA, and 10 mM EDTA. Following overnight incubation at 4°C, while rotating, the sepharose was washed three times with 1 mL PT and two times with 1 mL PBS. Precipitated proteins were eluted by addition of 50 µL 1× NuPAGE Sample buffer solution (Invitrogen) and incubation at 70°C for 10 min. After spinning down the sepharose, SDS-PAGE under non-reducing conditions was performed using a Novex NuPAGE 10 or 4–12% Bis–Tris gel followed by Western Blot onto a nitrocellulose membrane (Novex iBlot Gel Transfer kit, Invitrogen). Membranes were blocked with 1% (v/v) Western Blocking Reagent (WBR) (Roche, Basel, Switzerland) in PBS for 30 min and incubated with 1 µg/mL biotinylated polyclonal rabbit anti-FHR-3 in PBS + 0.5% (v/v) WBR, O/N. After washing three times with PT, membranes were incubated with 0.1% (v/v) Strep-HRP in PBS + 0.5% (v/v) WBR. After 1 h, the membranes were washed three times with PT followed by two washes with PBS. Western Blots were developed with the Pierce ECL 2 Western Blotting substrate kit (Thermo Scientific) according to the manufacturer’s instructions and analyzed using the ChemiDoc™ MP System (BioRad, Hercules, CA, USA).

### FHR-4A ELISA

To measure FHR-4A in serum, RM-19 was coated (3 µg/mL in PBS) onto Nunc Maxisorp 96-well microtiter plates (Invitrogen) by O/N incubation at room temperature. After coating, plates were washed five times with PT, followed by incubation with 1 µg/mL anti-FHR-4A.04 in HPE, for 1 h. After washing, samples, diluted in HPE, were added and incubated for 1 h. Following washing with PT, 0.5 µg/mL biotinylated polyclonal anti-FHR-3 (in HPE) was incubated on the plate for 1 h. Next, unbound conjugate was washed away and the wells were incubated with 0.01% (v/v) strep-poly-HRP (Sanquin), diluted in HPE, for 20 min. After washing, the assay was developed as described above using TMB and measuring absorbance at 450 nm and correcting for the absorbance at 540 nm. All ELISA steps were performed with a volume of 100 µL per well and incubated at room temperature while shaking unless stated otherwise. For the calibration of the FHR-4A ELISA, highly pure rhFHR-4A was used (Figure S1B in Supplemental Material) of which the concentration was determined by measuring the absorbance at 280 nm and using an absorbance coefficient of 2.134 (0.1% w/v solution).

### Statistical Analysis

Analysis and statistical tests were performed using GraphPad Prism, version 7.03 (GraphPad Software, La Jolla, CA, USA).

## Results

### Characterization of mAbs Against FHR-4A

We characterized 13 mouse mAbs raised against rhFHR-4A. All mAbs were first tested for cross-reactivity against all other members of the FH protein family (Figure 1A). The mAbs were named in order of increasing cross-reactivity: anti-FHR-4A.01 to 4A.06 being mono-specific for rhFHR-4A, anti-FHR-4A.07, and 4A.08 recognizing both rhFHR-4A and -4B, anti-FHR-4A.09 to 4A.11 recognizing rhFHR-3, 4A, and -4B, anti-FHR-4A.12 binding rhFHR-2, -3, -4A, and -4B, whereas anti-FHR-4A.13 recognizes all rhFHRs except rhFHR-5. None of the anti-FHR-4 mAbs showed cross-reactivity with either FHR-5 or FH.

With the use of recombinant fragments comprising different FHR-4A domains (Figure 1B), the epitope location of almost all anti-FHR-4A mAbs were mapped. As expected, all FHR-4A-specific mAbs (anti-FHR-4A.01 to 4A.06) bound to an epitope located in domains 2–5, which are unique for rhFHR-4A and not present in rhFHR-4B (Figures 1C,D). Of these FHR-4A specific mAbs, only anti-FHR-4A.04 recognized an epitope located in domain 5, whereas all other mAbs bound to an epitope in domain 2 or 3. Anti-FHR-4A.01, 4A.02, 4A.03, 4A.05, and 4A.06, competed with each other for the binding of rhFHR-4A, indicating identical or partially overlapping epitopes (data not shown).

Of the mAbs that cross-reacted with rhFHR-4B and other FHR protein family members, most appeared to bind to either of two epitopes in rhFHR-4A, reflecting the high degree of similarity between these SCR domains 2–5 in FHR-4A and the domains 1, 6, 7, and 8 in FHR-4A and FHR-4B.

Only anti-FHR-4A.09 appeared to have one epitope in domain 8 or 9, recognizing both rhFHR-4A and rhFHR-4B, while also cross-reacting with FHR-3.

The cross-reactive mAbs anti-FHR-4A.08 and anti-FHR-4A.11 were able to block binding of rhFHR-4A to the monospecific FHR-4A mAbs; however, in the reverse setting, this cross-blocking was not achieved (Figure 1E). This is in line with the presence of two epitopes for anti-FHR-4A.08 and 4A.11 in rhFHR-4A. It indicates that one of the two epitopes for anti-FHR-4A.08 and anti-FHR-4A.11 is partially overlapping or sterically hindering the binding site of the monospecific mAbs. The epitope location of anti-FHR-4A.13 could not be mapped due to a very low and ambiguous binding signal when the fragments were used. However, binding of rhFHR-4A to anti-FHR-4A.13 could be blocked with anti-FHR-4A.09, suggesting an epitope location in either domain 8 or 9 for anti-FHR-4A.13. An overview of the mAbs characteristics, the mapped epitope location, and the cross-reactivity is given in Table 1.

**Table 1:** Anti-factor H-related (FHR)-4A monoclonal antibodies characterized in this study.

		Epitope location (domain)			
Designation	Mouse isotype	1st	2nd	Cross-reactivity	Competes with
Anti-FHR-4A.01	IgG1, Kappa	2–3	–	None	4A.02, 4A.03, 4A.05, 4A.06
Anti-FHR-4A.02	IgG1, Kappa	2–3	–	None	4A.01, 4A.03, 4A.05, 4A.06
Anti-FHR-4A.03	IgG1, Kappa	2–3	-	None	4A.01, 4A.02, 4A.05, 4A.06
Anti-FHR-4A.04	IgG1, Kappa	5	–	None	None
Anti-FHR-4A.05	IgG2b, Kappa	2–3	–	None	4A.01, 4A.02, 4A.03, 4A.06
Anti-FHR-4A.06	IgG1, Kappa	2–3	–	None	4A.01, 4A.02, 4A.03, 4A.05
Anti-FHR-4A.07	IgG1, Kappa	2–3	6–7	rhFHR-4B	None
Anti-FHR-4A.08	IgG1, Kappa	1	5	rhFHR-4B	4A.01, 4A.02, 4A.03, 4A.05, 4A.06
Anti-FHR-4A.09	IgG1, Kappa	8–9	–	rhFHR-3, -4B	None
Anti-FHR-4A.10	IgG1, Kappa	4	8	rhFHR-3, -4B	None
Anti-FHR-4A.11	IgG1, Kappa	2–3	6–7	rhFHR-3, -4B	4A.01, 4A.02, 4A.03, 4A.05, 4A.06
Anti-FHR-4A.12	IgG1, Kappa	4	8	rhFHR-2, -3, -4B	None
Anti-FHR-4A.13	IgG1, Kappa	Unknown	Unknown	rhFHR-1, -2, -3, -4B	4A.09

### Only FHR-4A Is Detected in NHS

**Figure 2:**
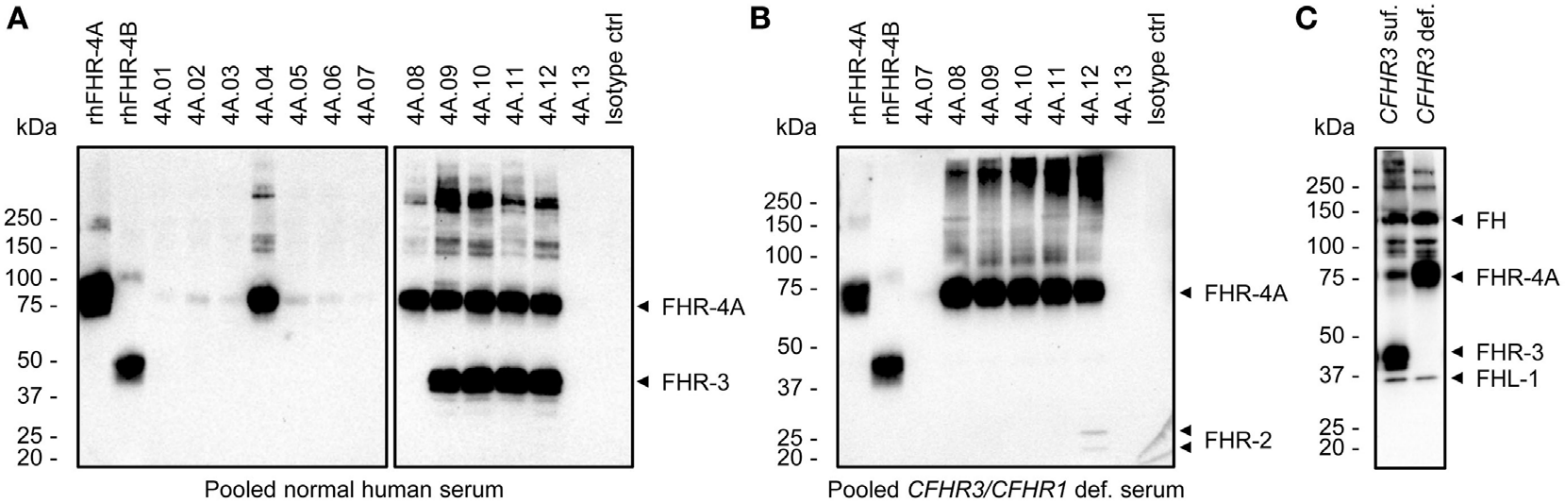
Immunoprecipitation (IP) of plasma-derived factor H-related (FHR) proteins by the anti-FHR-4A monoclonal antibodies (mAbs). **(A)** Western blot following IP from pooled normal human serum using the indicated mAbs. For comparison, 100 ng rhFHR-4A and rhFHR-4B were loaded on the left side of the gel. Precipitated proteins are indicated with arrowheads on the right side of the blot. **(B)** As in **(A)**, but with pooled serum of healthy donors who are deficient for *CFHR3* and *CFHR1* and using only the cross-reactive anti-FHR-4A mAbs for IP. **(C)** IP using polyclonal anti-FHR-3 and serum of two healthy donors with either two *CFHR3* (*CFHR3* suf.) or no *CFHR3* (*CFHR3* def.) gene copies. All Western blots were stained with cross-reactive biotinylated polyclonal anti-FHR-3. Precipitated proteins are indicated with arrowheads on the right side of each blot. Results are representative of multiple independent experiments.

Next, we tested whether the anti-FHR-4A mAbs capture FHR-4A and FHR-4B from NHS. To this end, an IP followed by Western Blot was performed, which was developed with cross-reactive biotinylated polyclonal anti-FHR-3. Of the specific anti-FHR-4A mAbs, only anti-FHR-4A.04 seemed to efficiently capture FHR-4A from NHS, resulting in a clear protein band corresponding to the molecular weight of FHR-4A (86 kDa) (Figure 2A). Only a very faint FHR-4A band was visible in the precipitates of anti-FHR-4A.01, 4A.02, 4A.03, 4A.05, and 4A.06. In addition, both anti-FHR-4A.07 and anti-FHR-4A.13 were neither able to efficiently immunoprecipitate FHR-4A from serum nor any of the other FHR proteins with which these mAbs cross-react. A band corresponding to FHR-4B (43 kDa) in the IP with anti-FHR-4A.08 also appeared to be missing, while the mAb was found to be cross-reactive with rhFHR-4B in the ELISA. A protein band corresponding to FHR-4B could not be distinguished in the IP of anti-FHR-4A.09 to anti-FHR-4A.12, as these mAbs also precipitated FHR-3 (43–50 kDa), which migrates at the same expected molecular weight and is also recognized by the polyclonal antibody. Therefore, to better visualize the IP of FHR-4B, an IP from a FHR-3-deficient serum pool was performed, using the cross-reactive mAbs. However, also in these conditions, no band corresponding to FHR-4B was detected in the IP of anti-FHR-4A.08 to anti-FHR-4A.12, which was unexpected given the cross-reactivity we observed with rhFHR-4B in ELISA (Figure 2B). On the other hand, anti-FHR-4A.12 did immunoprecipitate FHR-2 from NHS, confirming the cross-reactivity results obtained with rhFHR-2. As a control, the IP was repeated with the cross-reactive polyclonal anti-FHR-3 using serum of two healthy donors, one with two *CFHR3* gene copies, and one with no *CFHR3* gene copies. This resulted in the precipitation of FH, FHL-1, FHR-3, and FHR-4A (Figure 2C). However, also with this set-up, no FHR-4B was detected.

**Figure 3:**
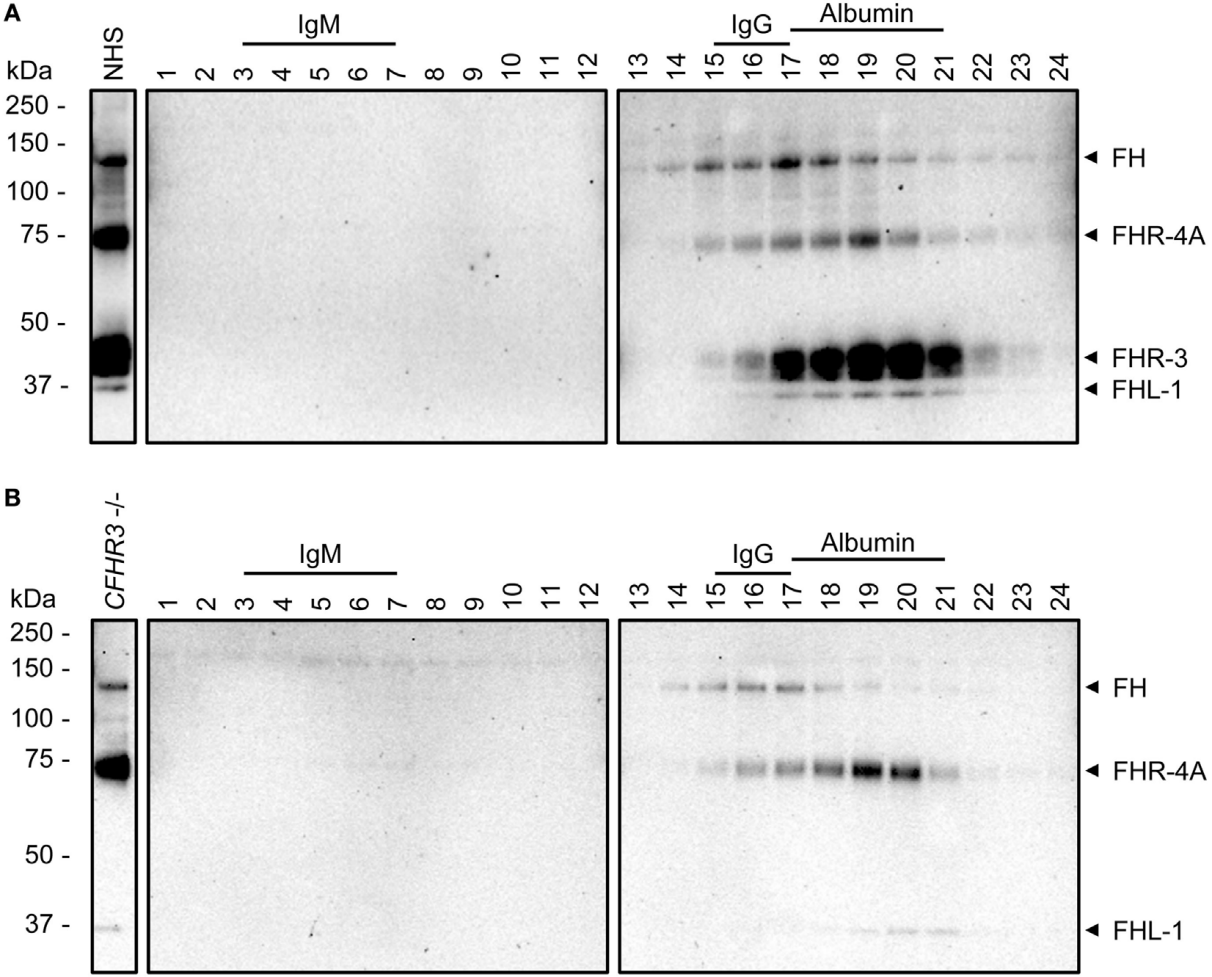
Sucrose gradients of normal human serum (NHS) and *CFHR3-*deficient serum. Western Blots of immunoprecipitation (IP) of fractions (1–24) from a sucrose gradient with **(A)** NHS or **(B)** a serum pool deficient for FHR-3 (*CFHR3*^−/−^). IP was performed using anti-FHR-3.3, Western Blots were stained with biotinylated polyclonal anti-FHR-3 antibody. Fractions containing IgM (970 kDa, fractions 3–7), IgG (150 kDa, fractions 15–17), or albumin (66 kDa, fractions 17–21) are indicated above the blots and used as size reference. Identified proteins are indicated with arrowheads on the right side.

Serum fractionated by sucrose gradients was previously used to investigate the molecular size of FHR proteins in their native state (18). Using a recently characterized anti-FHR-3 mAb that cross-reacts with FH, rhFHR-4A, rhFHR-4B, and FHL-1 (clone anti-FHR-3.3) (6), we investigated the circulating molecular size of FHR-4A relative to FH and FHR-3, by IP of these proteins from sucrose gradient fractions. As expected, FH was precipitated from the fractions also containing IgG, while FHR-3 was mainly present in the fractions containing albumin, corresponding with their respective molecular weights (155 and 50 kDa) (Figure 3A). FHR-4A was found in the fractions corresponding with its molecular weight (86 kDa), between the main fractions containing FH and FHR-3. Again, no bands corresponding with FHR-4B were found in the sucrose gradient fractions of neither the FHR-3 sufficient nor the FHR-3-deficient serum pool (Figure 3B).

### FHR-4A Levels Vary Greatly Between Individuals

**Figure 4:**
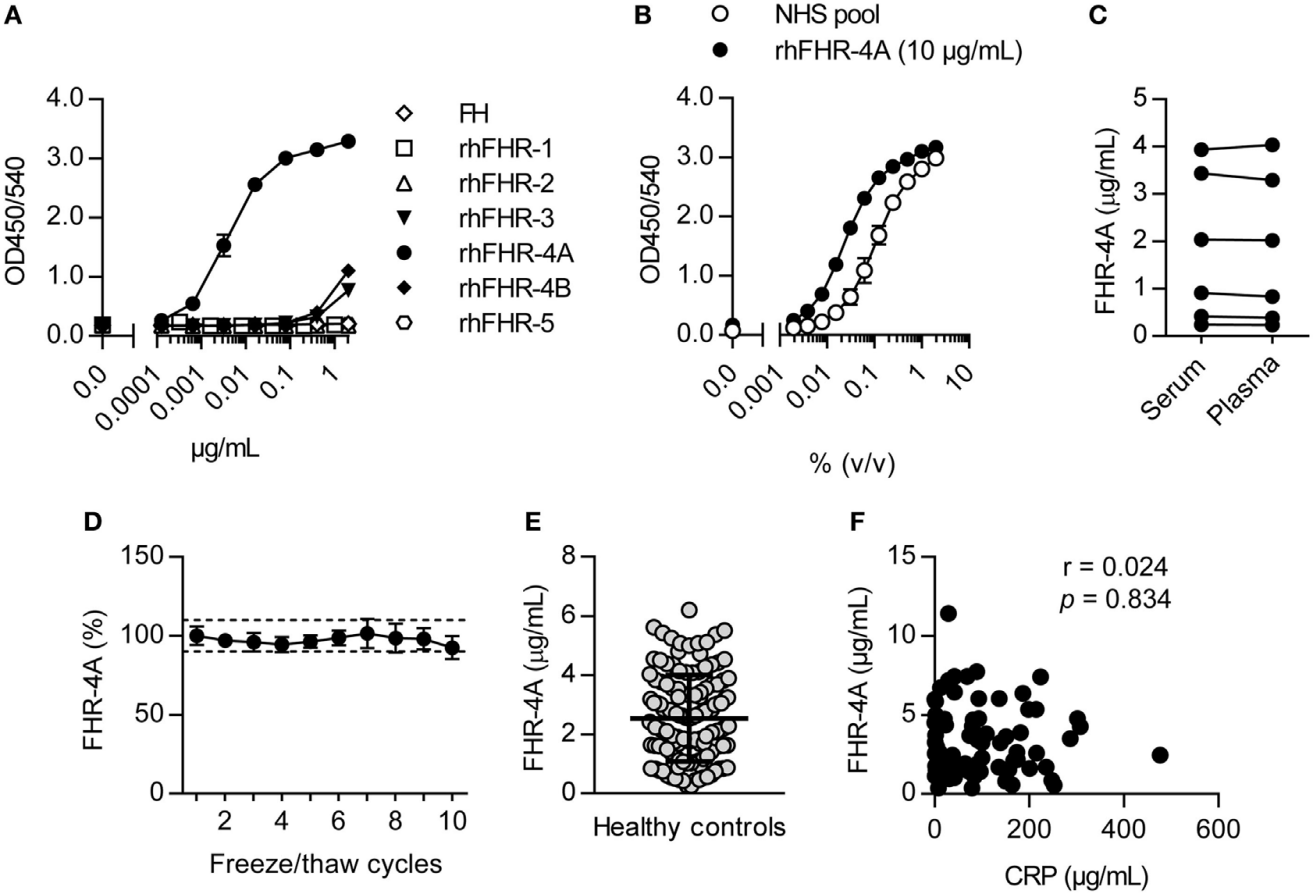
Development of the factor H-related (FHR)-4A specific ELISA. **(A)** Representative result on the reactivity of the FHR-4A ELISA against rhFHR proteins and plasma-derived FH. Anti-FHR-4A.04 was used as monospecific catching mAb, and binding of antigen was detected using cross-reactive polyclonal anti-FHR-3. **(B)** Calibration of pooled normal human serum using rhFHR-4A (10 µg/mL) in the FHR-4A ELISA. **(C)** Comparison of FHR-4A levels in seven paired serum and EDTA plasma samples using the FHR-4A ELISA. Each point represents the mean of three independent measurements per sample. **(D)** Effect of multiple freeze/thaw cycles. Each point represents the mean of three independent measurements per sample with error bars indicating SD. Dashed lines indicate 90–110% range. **(E)** Concentration of FHR-4A in 129 healthy donor sera, measured by ELISA. Each point represents the mean of three independent measurements per serum sample. Line indicates mean with SD. **(F)** Scatter plot of FHR-4A levels versus CRP levels determined in 78 patients with acute bacterial infection. Correlation was assessed using Spearman’s correlation test.

As we could not detect FHR-4B in human serum, we next focused on specifically measuring FHR-4A by ELISA. FHR-4A was measured using the monospecific mAb anti-FHR-4A.04, captured on immobilized RM-19, and using biotinylated polyclonal anti-FHR-3 antibody as a conjugate. The specificity of the FHR-4A ELISA was confirmed using rhFHR proteins and plasma purified FH, giving a clear signal for rhFHR-4A (Figure 4A). Both rhFHR-3 and rhFHR-4B were detected at the highest concentrations we tested, but the FHR-4A ELISA was found to be 843-fold and 1,490-fold more sensitive for rhFHR-4A compared to rhFHR-4B or rhFHR-3, respectively. Next, using purified rhFHR-4A, we calibrated a NHS pool to contain 2.33 µg/mL FHR-4A, which was subsequently used as a calibration curve in the FHR-4A ELISA (Figure 4B). FHR-4A levels in serum and plasma did not differ (Figure 4C, *P* > 0.05), nor were the levels affected by repeated freeze/thaw cycles (Figure 4D). Next, we measured the levels of FHR-4A in serum from 129 healthy donors. The average concentration of FHR-4A was found to be close to the concentration found in the NHS pool, being 2.55 µg/mL or 29.65 nM. Moreover, FHR-4A levels were found to be highly variable with a SD of 1.46 µg/mL and ranging from 0.26 up to 6.20 µg/mL (Figure 4E). We subsequently investigated whether FHR-4A behaves as an acute phase protein. To this end, we measured FHR-4A levels in 78 patients during an acute bacterial infection. No correlation was found for FHR-4A with CRP levels (*r* = 0.024, *P* = 0.834, Figure 4F). Of note, no total deficiency for FHR-4A was found in any healthy donor or patient measured during this study.

## Discussion

The FHR proteins are hypothesized to act as antagonists of complement regulator FH, competing for binding to ligands and allowing, instead of inhibiting, complement activation on surfaces (9). Several reports have shown that recombinant FHR-4A and -4B indeed bind to known FH ligands and allow complement activation to occur *in vitro* (11, 14, 15). To date, normal levels of FHR-1, FHR-2, FHR-3, and FHR-5 have been reported and were found to be circulating at much lower concentration as compared to FH (6, 18, 21, 22). In this report, we describe the circulating levels of FHR-4A in healthy individuals, using novel, well-characterized anti-FHR-4A mAbs. FHR-4B is apparently absent in blood, implying that FHR-4A is the dominant splice variant of *CFHR4*.

We obtained thirteen mAbs that were raised against rhFHR-4A, of which, six were found to be monospecific for FHR-4A. Their epitopes were mapped to be located within the four SCR domains unique for FHR-4A (and absent in FHR-4B). Of the seven cross-reactive mAbs that could also recognize the rhFHR-4B protein, six mAbs were found to bind to two epitopes within rhFHR-4A, corresponding to SCR domains that share high sequence similarity within rhFHR-4A because of the known internal gene duplication. The presence of two epitopes for these mAbs in rhFHR-4A was further supported by competition experiments. The rhFHR-4A-specific mAbs (one epitope) were not able to prevent binding of rhFHR-4A to cross-reactive mAbs (two epitopes), whereas the cross-reactive mAbs blocked binding to the specific mAbs, indicating that one of the two epitopes of the cross-reactive mAbs is overlapping with the epitope of the specific anti-FHR-4A mAbs.

Seven mAbs (anti-FHR-4A.01, -4A.02, -4A.03, -4A.05, -4A.06, -4A.07, and -4A.13) seemed unable or very inefficient in immunoprecipitating FHR-4A from NHS. The lack thereof is indicative for a much lower binding affinity for plasma-derived FHR-4A compared to rhFHR-4A. Strikingly, the epitopes of anti-FHR-4A.01, -4A.02, -4A.03, -4A.05, -4A.06, and -4A.07 are all located in SCR domains of FHR-4A that contain (multiple) putative *N*-linked glycosylation sites. While rhFHR-4A is produced by HEK293F cells and thus possesses glycans from human origin, it is possible that differences in the exact glycan composition from those found *in vivo* are involved in the apparent lack of binding affinity toward serum-derived FHR-4A. This might also be the case for anti-FHR-4A.13, for which we could not precisely map the epitope location.

We were unable to identify a band corresponding to FHR-4B in the IP by five different cross-reactive mAbs against rhFHR-4A and rhFHR-4B. These mAbs were able to precipitate FHR-4A from serum. Because anti-FHR-4A.08, -4A.10, -4A.11, and -4A.12 all recognized two epitopes in rhFHR-4A, it is possible that the lack of FHR-4B detection following IP was caused by a difference in binding avidity, favoring FHR-4A over FHR-4B. However, anti-FHR-4A.09, which also did not precipitate FHR-4B from human serum, only recognized one epitope in domain 8/9 or 4/5 of FHR-4A and FHR-4B, respectively. Thus far, only one report has suggested the presence of FHR-4B in human serum, being distinguished from FHR-3 by Western blotting following 2D electrophoresis separation (23). These blots were stained using cross-reactive polyclonal antibodies, making it unclear whether it is truly FHR-4B that was originally detected or not. Even if present, the levels of freely circulating FHR-4B must be extremely low compared to FHR-4A, since it was undetectable in any of our assays. Our current results are supported by previous reports, in which Western Blot analysis of human plasma also seems to indicate that FHR-4A is the dominant splice variant (8, 24). Hence, we developed an ELISA capable of specifically measuring FHR-4A and determined the normal average concentration of FHR-4A at 2.55 µg/mL (i.e., 29.65 nM). This is a 67.5-fold molar difference compared to the ~2 μM concentration of FH in human serum. The FHR-4A levels reported here are in line with previous reported levels determined by mass spectrometry (25) but stand in great contrast with the FHR-4 concentration of 25.4 µg/mL, as determined previously by an immunoassay (11). As discussed above, it seems unlikely that FHR-4B, which in theory was also measured in the previous report, can account for this difference. Both ELISAs catch FHR-4A with a monoclonal antibody while detecting it with a polyclonal antibody. Hence, it is more likely that there is a difference in the Sf9-derived rhFHR-4A previously used (14) and the HEK293F-derived rhFHR-4A used here for the calibration of the assays. Considering the recently reported normal values for all other FHR proteins (6, 18, 21, 22), ranging from 0.7 to 12 µg/mL (with FHR-1 being the most abundant FHR protein), it seems the values that we report for FHR-4A are likely to be correct.

We found considerable variation in FHR-4A concentration among healthy individuals, which is in line with previous observations by others (8, 17). This high degree of variation is also found in FHR-3 (6). However, the variation in FHR-3 levels can, to a major extent, be explained by the *CFHR3/CFHR1* deletion, which is relatively common with an allele frequency of about 20% in the Western population (12, 13, 26–30). The second known deletion in the *CFH-CFHR* locus, encompassing *CFHR1* and *CFHR4*, is far less common with an allele frequency of about 0.9% in the Western population (31). Thus, it is unlikely that CNV of *CFHR4* explains the variation seen in FHR-4A levels, suggesting that other as yet unknown genetic variations determine the concentrations of FHR-4A, which is independent of inflammation since FHR-4A did not behave as an early acute phase protein. Studies investigating the genetic variation(s) resulting in altered FHR-4A expression are currently ongoing.

In summary, we characterized novel anti-FHR-4A mAbs, which were employed to develop a highly specific FHR-4A ELISA. FHR-4A was found to be the dominant splice variant of *CFHR4* and circulates in healthy individuals at an average concentration of 2.55 (±1.46) μg/mL. Similar to the other FHR proteins, the circulating levels of FHR-4A are much lower as compared to FH (~0.03 versus ~2 μM, respectively).

## Ethics Statement

Healthy donor serum samples were collected as part of a previous study from anonymous, healthy volunteers with informed, written consent in accordance with Dutch regulations, and this study was approved by the Sanquin Ethical Advisory Board in accordance with the Declaration of Helsinki.

## Author Contributions

RP, DW, and TK designed the study. RP, MJ, DW, and TK designed experiments. RP, MB, and AvB performed experiments. All authors analyzed and discussed data. RP, DW, and TK wrote the first draft of the manuscript. All authors revised the data and contributed to the final version of the manuscript.

## Conflict of Interest Statement

The authors declare that the research was conducted in the absence of any commercial or financial relationships that could be construed as a potential conflict of interest.
