# Supplementary material for: Complement Factor H-Related Protein 4A Is the Dominant Circulating Splice Variant of CFHR4
Source: Front Immunol. 2018 Apr 17;9:729. doi: 10.3389/fimmu.2018.00729 (PMC5913293; doi:10.3389/fimmu.2018.00729)
Supplement: Supplementary file 1 [file image_1.PDF]

Pouw RB, Brouwer MC, van Beek AE, Józsi M, Wouters D and Kuijpers TW (2018) Complement factor H-related protein 4A is the dominant circulating splice variant of *CFHR4*. *Front. Immunol. 9:729. doi: 10.3389/fimmu.2018.00729*

Supplemental materials

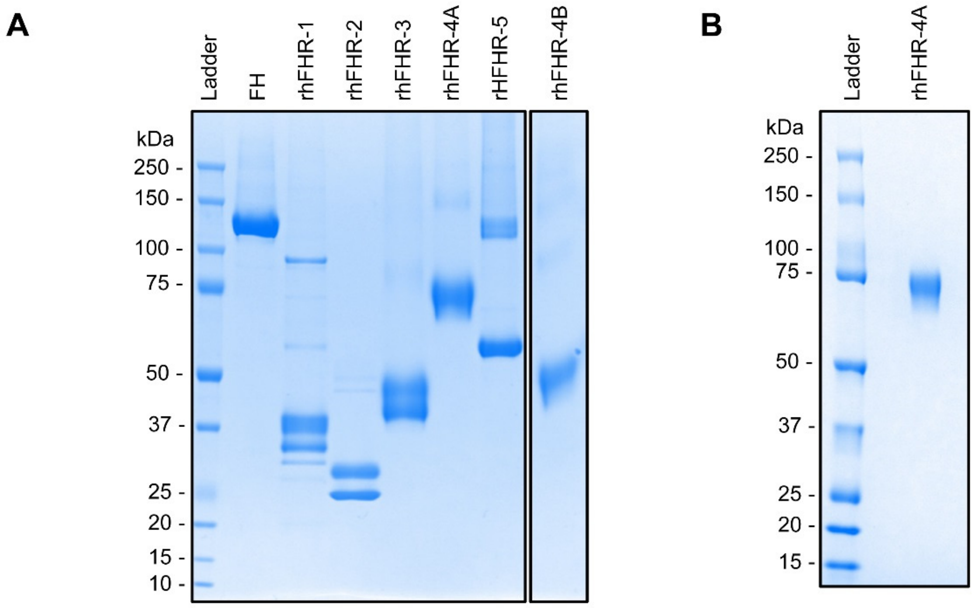

Supplemental figure 1. Recombinant FHR proteins used in this study. SDS-PAGE gels stained with PAGEBlue showing the rhFHR proteins after Ni^{2+} chromatography purification (**a**) and rhFHR-4A used for the calibration of the FHR-4A ELISA (**b**). High molecular weight impurities in rhFHR-4A were removed using a 100 kDa molecular weight cut-off Amicon® filter. Ladder indicating the molecular weight in kDa is shown on the left side of the gels.
